# Tumors of the Jaws

**Published:** 1908-03-15

**Authors:** M. Schamberg


### TUMORS OF THE JAWS.

BY M. SCHAMBERG, D.D.S.

In dealing with tumefactions about the face, the first step is to determine whether the condition is an inflammatory swelling brought about by an alveolar abscess, impacted molar, etc. This is not always an easy task. Only this evening did a patient appear at my office, with an impacted third molar which was causing the same distortion of the face as was present in a case of mandibular sarcoma, for whom I removed a large section of the jaw, one week ago. Cases of extensive osteomyelitis must first be differentiated from malignant growths of the jaw, for they frequently present a similar appearance. I have operated upon a number of cases of osteomyelitis where a complete curettement of the entire inner portion of the jaw was necessary, leaving but a shell of bone behind, to fill in with granulation tissue, the operation being performed from within the mouth. An osteomyelitis, extending over a period of six months, or a year, might readily be mistaken for a tumor of malignant nature, which would naturally call for a more extensive and disfiguring operation. One of the most important aids in my practice, to an accurate diagnosis of these obscure cases, is the radiograph. Some of you doubtless recall the value of radiography, in the case of the encysted superior cuspid that I operated upon before the First District Dental Society, last year, at the Hospital for Ruptured and Crippled. I was glad to have present on that occasion, a number of prominent surgeons, who saw the evidence of the knowledge which was imparted by the radiograph and which impelled me to extend the bone cutting, until the tooth distinctly shown in the picture, was exposed and removed.

There is a great tendency on the part of many practitioners to overlook the possibility of initial lesions of syphilis appearing in the mouth. I have seen several cases of extra-genital chancres about the mouth, acquired in an innocent way by persons who never suspected the trouble, nor were they apprised of the fact, and some of them contemplated operation upon the lesion believing the condition to be cancer. There is ofttimes a marked similarity of appearance between an incipient epitheleomia and a chancre when these lesions appear upon the mucous membrane of the mouth and lips.

It is important that an early diagnosis be made of all malignant growths about the mouth, for in this region they develop rapidly and terminate fatally unless operated upon with thoroughness during their incipiency. Within the past few weeks I was called upon to diagnose several cases of buccal carcinoma which had passed to the inoperable stage through failure of those in attendance upon the cases to recognize the character of lesion they were treating. The nature of these lesions was evident to me upon sight, for they presented the characteristic cauliflower-like formation which, when once seen about a deep irregular ulcer, leaves a lasting impression. In all cases, however, it is well to fortify the diagnosis by a microscopical examination of a portion removed from the tumor.

Acute inflammatory swellings usually reach a crisis within a week or ten days. Anything that persists beyond that time should be looked upon as a growth that demands closest scrutiny. If the tumefaction affects the outline of the jaw itself, and does not appear to involve the soft tissue, a radiograph is indicated to determine the true nature of the inner structure of the bone. It will assist in differentiating between encysted teeth, multilocular cysts, ostemyelitis, and sarcoma.

Tumors arising on the surface demand microscopical examinations whether they be small epuli or extensive growths, for most malignant lesions begin as the benign ones do, in a small way. The microscope offers the surest means of determining the true character of a tumor.

I believe that all epuli are deep-seated in origin and that they come from either the periosteum or peridental membrane. In most all that I have removed the base was found to be attached to the periosteal or peridental membrane, so that I usually remove the adjacent teeth.—*The Journal.*

